# Medical Countermeasures against Ricin Intoxication

**DOI:** 10.3390/toxins15020100

**Published:** 2023-01-20

**Authors:** Christine Rasetti-Escargueil, Arnaud Avril

**Affiliations:** 1 Unité des Bactéries Anaérobies et Toxines, Institut Pasteur, 25 Avenue du Docteur Roux, 75015 Paris, France; 2 Unité Immunopathologies, Département Microbiologie et Maladies Infectieuses, Institut de Recherche Biomédicale des Armées, 91220 Brétigny-sur-Orge, France

**Keywords:** antitoxin, antibodies, ricin, small-molecule inhibitors, medical countermeasures, vaccines

## Abstract

Ricin toxin is a disulfide-linked glycoprotein (AB toxin) comprising one enzymatic A chain (RTA) and one cell-binding B chain (RTB) contained in the castor bean, a *Ricinus* species. Ricin inhibits peptide chain elongation via disruption of the binding between elongation factors and ribosomes, resulting in apoptosis, inflammation, oxidative stress, and DNA damage, in addition to the classically known rRNA damage. Ricin has been used in traditional medicine throughout the world since prehistoric times. Because ricin toxin is highly toxic and can be readily extracted from beans, it could be used as a bioweapon (CDC B-list). Due to its extreme lethality and potential use as a biological weapon, ricin toxin remains a global public health concern requiring specific countermeasures. Currently, no specific treatment for ricin intoxication is available. This review focuses on the drugs under development. In particular, some examples are reviewed to demonstrate the proof of concept of antibody-based therapy. Chemical inhibitors, small proteins, and vaccines can serve as alternatives to antibodies or may be used in combination with antibodies.

## 1. Introduction

### 1.1. Generalities

Ricin toxin (RT) is produced in the endosperm of the seeds of *Ricinus communis* (*L. Euphorbiaceae* family), also called the castor bean or Palma Christi. The plant originally comes from Africa and Asia but is nowadays widespread throughout temperate, subtropical, and tropical areas. The castor bean grows as an invasive plant or is cultivated as an ornamental plant or for industry [1]. Depending on the *R. Communis* cultivars, the ricin belongs to a gene family that encodes up to seven ricin or ricin-like proteins, with isoforms D and E and the agglutinin RCA120 being the most abundant in the seeds. Ricin D and E are present in the seeds in different proportions depending on the cultivar and the seed of the plant. The seeds also contain ricinine, an alkaloid that is frequently used as a marker of ricin intoxication. 

RT is a disulfide-linked heterodimeric glycoprotein (AB toxin) composed of the Ricin Toxin A (RTA) chain and the Ricin Toxin Binding subunit B (RTB) chain. RTA is a N-glycosidase that cleaves a single adenine residue at the adenine position 4324 from the 28S ribosomal RNA loop within the 60S ribosomal subunit. This cleavage irreversibly inactivates the ribosome and protein synthesis. RTB is a galactose-specific lectin that binds onto glycolipids and glycoproteins of vertebrate cell surfaces and mediates the RTA translocation into the cytosol. RCA120 is a lectin that shares a sequence identity of 93% with the RTA chain and 84% with the RTB chain, but it is 50–2000 times less toxic than ricin. A single molecule of ricin is able to inactivate 1000–1500 ribosomes per minute [2,3]. RT is extracted from castor beans, representing 3–5% of their dry weight [4]. The mean weight of a ricin seed is ~3.5 g (ranging from 1.3 to 9.9 g depending on the species), and each seed contains ~9.3 mg of ricin per gram of seed (from 1.6 mg to 32 mg per gram of seed). It is estimated that the ingestion of three seeds for children or from four to eight seeds for adults is generally lethal [5]. RT can be purified by sodium hydroxide precipitation (crude preparation) or using chromatography after one initial extraction step from the castor bean. However, RT is highly toxic, even when pulverized, and can be readily used as a bioweapon. The ubiquity of castor beans, ease of extraction, and ricin’s chemical stability make ricin an attractive and inexpensive agent to be produced in large quantities by individuals or nations. The human mean lethal dose (LD_50_) of RT varies according to the route of exposure. It is estimated to be between 2 and 10 µg·kg^−1^ through aerosol or parenteral administration and between 1 and 20 mg·kg^−1^ through ingestion [4,6,7].

### 1.2. Ricin Use in Traditional Medicine

The culture of castor beans and their manufacture are mainly localized in a small geographic region of Gujarat in Western India, but also, to a much lower extent, in other countries such as Mozambique, Brazil, and China. Castor oil accounts for only 0.15% of the production of vegetable oils in the World. However, worldwide consumption of castor oil has increased by more than 240% during the past 30 years, rising from 400 tons in 1985 to 2050 tons in 2020, even though the use of ricin-derived products has decreased in more developed countries. In 2020, India produced 1840 tons out of 2050 tons. Processing of castor beans generates two main byproducts: the oil from one side and the husks and ricin meal/cake from the other side. Castor oil and castor cake can be produced from the cold pressure of ricin seeds. The oil is viscous, pale yellow, nonvolatile, and nondrying. Castor beans have been used in traditional medicine throughout the world: on the Mediterranean coasts, they have been used for their galactagogue properties; in Africa, castor beans have been used to promote lactation, for various articular, cutaneous, or ocular diseases, or as an abortive; in the Caribbean, they have been used to cure erysipelas, flu, inflammation of the womb, and stomach aches; in Brazil, they have been used as an anthelmintic or a purgative, or they have been locally applied to treat hair loss and heal wounds or burns (reviewed in [8]). The activities of castor seeds are due to the activation of smooth-muscle cells through the stimulation of prostaglandin EP3 receptors by ricinoleic acid [9]. The purgative actions of castor seeds are due to their irritating effect on the intestinal cells [10].

As evidenced by archaeological findings, the castor plant has been known since immemorial times, and its use dates to the prehistoric era. Ricinoleic and ricinelaidic acid traces were found in wax on a wooden poison applicator, dating back to about 24,000 years ago [11]. Entire chapters are dedicated to the pharmacology of the castor bean in the ancient Egyptian Ebers or Hearst Papyrus, dating back to before 1500 BCE. Castor bean is indicated as an abortifacient, a laxative, and a remedy for abscess illness or baldness, as well as an ingredient in prescriptions to expel fluid accumulation or promote diuresis [12,13]. Around 400 BCE, Hippocrates prescribed castor bean oil as a laxative and for its detoxifying action [14]. Similarly, Pedanius Dioscorides (40–90 CE), the Greek herbalist and physician in “De Materia Medica”, and Pliny the Elder (23–79 CE) in “Naturalis historia” described the expectorant, diuretic, emetic, laxative, and anti-inflammatory uses of castor beans [15,16]. In Chinese traditional medicine, castor beans are also recommended for their anthelmintic properties or to treat ulcers and chronic wounds [17]. In Ayurveda medicine, the castor plant is recommended for rheumatic conditions, gastropathy, constipation, inflammation, fever, bronchitis, cough, skin diseases, colic, and lumbago [18]. 

Although RT has been traditionally used as an herbal medicine, its modern uses, unfortunately, also encompass its exploitation as an easily produced bioweapon.

### 1.3. Symptoms

Some symptoms induced by ricin intoxication are similar, whatever the route of intoxication and their severity is dose-dependent. However, some symptoms are more specific to the route of intoxication. Symptoms generally arise 2–6 h after ingestion and within 8 h after inhalation, but symptoms may sometimes be observed after 20 h. Physical symptoms are abdominal pain, emesis, diarrhea with or without blood, muscular pain, cramps in the limbs, circulatory collapse, dyspnea, and dehydration. Muscular pain and circulatory collapse are more commonly observed with injected ricin, as well as pain at the injection site. When exposed to an aerosol of ricin, redness and pain in the skin and the eyes may be observed. Respiratory distress (difficulty breathing), fever, cough, nausea, and tightness in the chest are also observed. Heavy sweating may follow as well as fluid building up in the lungs (pulmonary edema). Biochemical analyses often reveal an increase in white blood cells, blood urea nitrogen, aspartate aminotransferase, and alanine aminotransferase, indicating dysfunction of the liver and kidneys. Autopsies in fatal cases have shown hemorrhagic necrosis in the intestines and heart and edema in the lungs.

### 1.4. Industrial Use of Castor Oil Derivatives

The castor oil offers interesting properties due to its unique structure, making it appropriate for various industrial applications such as the manufacture of biofuels, soaps, waxes, greases, coatings, and, in particular, lubricants [19]. However, industrial uses, such as the use of waste products from castor oil processing as fertilizers, pose a greater risk of exposure to ricin.

Lubricants, hydraulic, and brake fluids: one of the main utilizations of castor oil is the synthesis of acyloxy castor polyol esters to produce low pour point lubricant. The resulting lubricant is to be a smoke pollution reducer (50–70% smoke reduction at a 1% oil–fuel ratio) and could be biodegradable. 

Fertilizers: After cold pressure of castor beans, husks and ricin meal/cake are also produced. For each ton of castor oil, 1.31 tons of husks and 1.1 tons of ricin meal/cake are obtained. The ricin meal/cake is an organic fertilizer with high nitrogen and phosphorus content that is used worldwide by gardeners. Even if ricin is not toxic by the cutaneous route of exposure, the fertilizer must be inactivated. A range of physical, chemical and biological treatments have been used for ricin inactivation. Nevertheless, it was proved that this inactivation could not be complete and that remaining toxicity is possible. 

Fuel and biodiesel: Methyl esters of castor oil can be produced by the transesterification method. This methyl ester can be used as a fuel additive to obtain biodiesel, which is a viable alternative to pure diesel [20,21].

Polymer materials: Biodegradable polyesters represent one of the most common applications using castor oil. They are biodegradable and can be used for several applications in the biomedical field, including the preparation of elastomers or packaging materials. Castor oil can also be combined with other monomers to produce copolymers used for solid implants or in situ injectable hydrophobic gel.

Coatings: Finally, when castor oil is dehydrated by nonconjugated oil–maleic anhydride adducts, it could be used as coatings or paints.

## 2. Exploitation of Ricin as a Bio-Weapon

Due to its availability, toxicity, ease of production, and lack of curative treatments, ricin has been classified by the Centers for Disease Control and Prevention (CDC) as a category B biological weapon and is a prohibited substance by both the Chemical Weapons Convention (CWC, schedule 1 compound) and the Biological Weapons Convention (BWC). Ricin is the principal example of a CDC category B agent that could be used as a bioweapon. In particular, ricin possession or purification is strictly regulated and controlled by the Organization for the Prohibition of Chemical Weapons (OPCW). Small-scale events involving the spread of RT in civilian populations could lead to panic and severe economic disruption, even if RT is 100-fold less toxic than botulinum toxin. 

Although ricin toxin is not infectious and its LD_50_ is higher than that of other toxins, it is easy to extract from *Ricinus communis* seeds, and several terrorists have successfully purified it. An RT extract can be easily produced from castor bean seeds, and therefore, recipes have been circulated on the internet in manuals that may assist potential terrorists. However, thanks to the low content of toxin in the final extracts, recipes in terrorist “cookbooks” would be unable to cause mass casualties in the case of ricin, whatever the exposure route [22].

Several potential utilizations of ricin as a biological weapon have been reported. Ricin was weaponized during World War Two by at least the USA and the United Kingdom under the appellation of “compound W”. The most famous utilization of ricin as a biological weapon was during the assassination of Georgi Markov, a Bulgarian dissident, in London in 1978. A micro-engineered pellet that might have contained ricin was shot in the leg of Georgi Markov with an umbrella wielded by someone associated with the Bulgarian secret service. During the United Nations’ inspection of Iraq in 1990, after the Gulf War, it was observed that Iraq would have produced 10 L of ricin for military purposes. In 2001, ricin was produced in France by the “Al-Qaeda chemist”, and in 2003, vials containing ricin traces were found in a French train station. In 2004, 2013, and 2018, letters and parcels containing ricin were sent to Senator Bill Frist, Barack Obama, Donald Trump, and the Pentagon. In 2018, a Lebanese man who was part of Daesh was arrested in Italy. He was suspected of planning the contamination of water tanks with ricin or anthrax. In 2018, in Cologne, the German police arrested Sief Allah H., a Tunisian who was part of Daesh, because he was manufacturing an explosive device containing ricin [23]. During the arrest, 84.3 mg of ricin and ~3300 ricin seeds were seized. Explosive engines containing ricin were produced by the suspect. This long history of ricin as a biological weapon underlines the need for prophylaxis and therapies. 

## 3. Diversity and Structure of Ricin

### 3.1. Ricin Diversity and Structure

Contrary to ricin E, ricin D is present in all castor bean seeds. For instance, ricin E is not found in *R. communis cv. zanzibarensis*, which is known to contain only the ricin D isoform, but it is found in *R. communis cv. Carmencita*. Moreover, differences in post-translational modifications (e.g., N-glycosylation), which can also impact toxicity, have been described depending on the cultivar and origin of the seed from which ricin is isolated. The ricin E has been defined as a hybrid form of ricin because it is composed of a ricin-like A chain, the N-terminal half of the B chain and an RCA-like C-terminal half of the B chain [24,25]. Initially, it was considered that ricin E was present in small-grain castor beans, but a more recent publication questioned this [24]. “Small seeds” refer to cultivars where 100 seeds weigh less than 20 g.

The bi-chain nature of the ricin structure was demonstrated in the early 1970s, when ricin was found to be composed of an active chain (A chain) and a binding chain (B chain) linked by a disulfide bond [26,27]. The primary sequences of both ricin chains and their holotoxin structures at 2.8 Å resolution were solved soon after [28,29].

The refined ricin structure at 2.5 Å revealed that the ricin A chain is a globular protein consisting of 267 amino acids organized into eight α-helices and eight β-strand structures. The ricin B chain consists of 262 amino acids having two homologue domains, each containing a lactose binding site [30,31,32,33]. It was then discovered that the ricin A chain can be cross-linked to the ribosomal proteins L9 and L10e [34,35] and that its active site involves key residues such as Tyr80, Tyr123, Glu177, Arg180, and Trp211. Ribosomal adenine is deemed to be trapped between Tyr80 and Tyr123 in a π stacking interaction and then protonated by Arg180, promoting the formation of an oxocarbenium moiety on the ribose [36,37]. 

### 3.2. Ricin Enzymatic Activity

By disrupting the binding between elongation factors and ribosomes, ricin inhibits peptide chain elongation, avoiding the elongation-factor-dependent GTPase activity [38,39]. Similar plant proteins have been identified to inhibit protein synthesis, holding a comparable polypeptide chain to the A chain of ricin: they are called “ribosome-inactivating proteins” (RIPs) [40,41]. Finally, in 1987, Endo et al. discovered the enzymatic nature of the ricin A chain, which cleaved the N-glycosidic bond of an adenine residue in rats, making RNA highly susceptible to hydrolysis [42]. Furthermore, ricin was found to release adenine from rRNA, DNA, and poly(ADP-ribosyl)ated poly(ADP-ribose) polymerase, an enzyme involved in DNA repair [43]. It was also highlighted that, in tandem with protein synthesis inhibition and apoptosis, ricin could act directly on DNA in several cellular models.

### 3.3. Ricin Cellular Uptake, Routing, and Toxicity

Early studies demonstrated that the ricin interaction with the cell starts with the binding of the B chain to galactosyl residues of both glycolipids and glycoproteins on the cell surface, allowing access to the endosomal compartment [44]. Ricin is subsequently internalized, using various endocytic pathways, to reach the Golgi apparatus and intoxicate the cell. Ricin first penetrates the cell via clathrin-dependent endocytosis [45]. Ricin is then delivered to early endosomes. In parallel, ricin is recycled back to the cell surface or delivered to lysosomes via late endosomes for proteolytical degradation [46,47]. Eiklid et al. showed that cell death induced by ricin is directly proportional to the ricin concentration. The velocity of ricin activity is much greater than the speed of re-synthesis/repair of ribosomes, and ricin’s half-life is sufficient to inactivate all ribosomes in the cells. These observations suggest that one molecule of active ricin that reaches its substrate could be enough to kill one cell [48]. In addition to the disruption of protein synthesis, it was discovered that ricin, and related toxins, may be retrogradely transported across neuronal processes, paving the way for innovative research in neurobiology [49]. Ricin is one of the most toxic plant toxins discovered with an IC_50_ of less than 0.1 to 1 pM. Considering their polynucleotide-depurinating activity, RIPs may have a wider toxic action such as the oxidative stress potential facilitation. This suggests that ricin is able to drive the induction of more than one cell death pathway [50].

However, the cellular mechanism of action that kills the cell is quite complex. Several studies show that the inhibition of protein synthesis is not always correlated with long-term ricin toxicity. It is now admitted that, in addition to the rRNA damage, ricin can induce apoptosis, inflammation, oxidative stress, and DNA damage. At present, the correlation between these processes is under investigation. In addition to expanding investigations on intoxication processes via intracellular transport, knowledge of ricin’s action on cells, including its ability to enter the cytosol, can be applied to treat infectious diseases or for even wider therapeutic purposes [51].

## 4. Detection of Ricin Toxin 

The detection of ricin is performed in various sample types, including body tissues and many other materials, to determine whether a person has been exposed to this agent prior to any therapeutic medical treatment. Moreover, from a legal and security viewpoint, it is essential to determine ricin exposure to attribute responsibilities and determine potential terrorist acts (see Table 1).

In the animal model, it was observed that the distribution of ricin in the body after intoxication is largely specific to the exposure route. It was initially observed that intact ricin is distributed in the liver, blood, lungs, spleen, kidneys, and heart [52]. Examination of urine samples withdrawn from the bladder with a syringe revealed that ricin excreted in urine was in the form of a low-molecular-weight degradation product. In an oral ingestion model, it was found that ricin excreted in the stool was biologically active in a cytotoxicity assay, providing evidence that intact ricin is present in feces [53]. Conversely, it was observed that intravenously administered ricin did not enter the gastrointestinal tract and, therefore, was not detected in feces. Therefore, the detection of ricin is impossible in hospital routines using urine samples with, for example, lateral flow assays.

The detection of ricin starting from other biological samples is more complex with laboratory techniques such as liquid chromatography–tandem mass spectrometry (LC-MS/MS). The difficulties are mainly due to the fact that ricin is a large heterogeneous protein with glycosylation. Nevertheless, mass spectrometry is essential for the differentiation between biologically active and inactive ricin, which is the key to evaluating the lethality of a bioterrorism threat and monitoring site decontamination. Ricin enrichment has been proposed with the mass spectrometry method to optimize sensitivity using ricin-specific antibodies [54]. However, the mass spectrometry assay format alone is unable to confirm functionality, and therefore, confirmation is needed to add a functional endpoint. To achieve functional confirmation, liquid chromatography and multiple reaction monitoring (MRM) mass spectrometry (MS) were combined with antibody-based affinity capture to quantify the amounts of ricin and RCA in extracts prepared from the seeds of eighteen cultivars of *R. communis*. In addition, the rRNA N-glycosidase activity of each cultivar was measured in comparison to one ricin standard [24]. The mass spectrometry assay format was further optimized using an in vitro matrix-assisted laser desorption/ionization (MALDI) time-of-flight (TOF) mass spectrometry (MS)-based activity assay that detects ricin-mediated depurination of synthetic substrates through optimization of the substrate, reaction conditions, and sample preparation. Using optimized parameters, a limit of detection of 0.2 ng·mL^−1^ of ricin spiked in the buffer and milk was achieved, representing an enhancement of more than 2 orders of magnitude in the assay sensitivity, allowing the detection of active ricin with an enhancement of 3 orders of magnitude in the dynamic range [55]. An innovative ricin capture method of magnetic beads coated with 4-aminophenyl-1-thiol-β-galactopyranoside has been adapted for use on a simple, benchtop MALDI-TOF MS mass spectrometer common in clinical microbiology laboratories. This assay format was found to be highly selective with no cross-reactivity from near neighbors and highly specific, with a panel of 19 cultivars all testing positive, thus allowing a confirmatory routine detection method for ricin in clinical laboratories lacking sophisticated mass spectrometers [56].

Immunodetection methods, such as enzyme-linked immunosorbent assays (ELISAs) and lateral flow assays, as well as kits for biological specimens and environmental samples, are largely described in the literature for their excellent performance in terms of specificity, simplicity, and analysis time [57]. Assay times were subsequently reduced using fluorescence-based fiber optics, colloidal gold particles, or electrochemiluminescence, with preserved sensitivity [58,59,60]. In addition, several ELISAs were developed to directly detect ricin in biological and environmental samples, with a limit of detection up to 2 pg·mL^−1^ [61,62]. Furthermore, ricinine is a good urinary biomarker of ricin intoxication that can also be detected in the serum [63,64]. Improvements in sensitivity were obtained using microspheres, also providing improvements in the assay time, as well as the use of magnetoelastic sensor surfaces [65,66]. Toxin capture antibodies can be replaced with DNA/RNA aptamers, single-domain antibodies, or sugar-conjugated materials [67,68,69] to improve reagent stability. The assay specificity and sensitivity were significantly improved when enrichment steps were coupled to enhanced detection technologies, such as surface plasmon resonance (SPR), polymerase chain reaction (PCR), or mass spectrometry (MS) [70,71,72,73,74,75,76]. Despite the many advantages of immunochemical methods, their inability to distinguish between biologically active and inactive RT precludes their use to assess functional impairment in case of a bioweapon threat.

The measurement of free adenine release provides a functional alternative to assess the catalytic activity of RT. The release of free adenine from ribosomes or from DNA and RNA substrates can be quantified using reverse-phase HPLC coupled with UV, MS, or fluorescence detection [77,78,79]. Other RTA activity assay methods use [^3^H]-adenine quantified with liquid scintillation counting, colorimetric enzyme reactions, or chemiluminescence [80]. RTA enzyme activity assays can be coupled to ricin-specific sample enrichment steps, allowing the detection of RT in parallel to A-chain activity assessment [81,82]. Ricin-specific RNA aptamers and carbohydrate compounds can be used for the recovery of ricin in suspected samples [67,83].

A robust cell-based assay that can detect ricin cytotoxicity can be used as a confirmatory test using sensitive fluorescence- or luminescence-based methods to measure cellular cytotoxicity [84]. Digital holographic microscopy was employed to show the morphological changes in cell lines intoxicated with ricin and abrin toxins. Ricin and abrin caused a decrease in HeLa and Vero cell confluence with decreased cell divisions and total cell counts, in parallel to an increased optical thickness and roughness compared to control cells. The addition of neutralizing antibodies against ricin or abrin inhibited those detrimental effects, showing the high sensitivity of the digital holographic microscopy method. This technique allows the early detection of active toxins [85].

In addition to functionality assessment, the first responders need reliable, easy-to-use, and sensitive methodologies for on-site detection of the toxins to guarantee a prompt response. Therefore, several on-site immunoassay platforms have been developed for mobile or on-site multiplex detection of security-sensitive toxins such as RT. Anti-ricin single-domain antibodies (sdAbs) have been isolated and derived from llamas, engineered to increase their thermal stability and to develop more sensitive immunoassays to detect RT [86].

In addition to toxin detection from a medical perspective, PCR can be used to detect DNA or RNA relevant for determining the cultivar, which is essential for forensics. Direct RNA sequencing, using the technology developed by Oxford Nanopore Technologies (ONT), can be used to detect a change in charge in the sarcin–ricin loop to identify depurination in samples exposed to ricin. The depurination measured is specific to ricin activity, as shown with neutralizing antibodies. This direct RNA sequencing technique confirmed the potential of the ONT technology to detect and quantify depurination events caused by RT. The new RIPpore assay format is of high importance to assess new inhibitors and for routine diagnostics of ricin exposure [87].

Ricinine (3-cyano-4-methoxy-N-methyl-2-pyridone) is a piperidine alkaloid (MW = 164.2 g·mol^−1^) that can be found in all parts of the plant, including in the seeds (~0.2–0.8% of the seed weight) and both in castor oil and ricin cake [88]. Ricinine is far less toxic than ricin; however, it can still induce hyperactivity and death by respiratory arrest at high doses in mice. In a mouse model, the LD_50_ was estimated to be 340 mg.kg^−1^ via the intraperitoneal injection (i.p.) route and 3 g·kg^−1^ orally [89]. It could be used as a strong insecticide, being soluble in water up to 2.7 mg·mL^−1^ at 10 °C. Ricinine is the best urinary biomarker of ricin intoxication and can also be detected in the serum [63,64]. Its detection in urine is an easy, cheap, and fast way to diagnose ricin intoxication. Because ricinine is unique to *Ricinus communis*, its presence implies exposure to a product derived from castor seeds that may include extracted ricin, which is helpful for both therapy and forensics. 

## 5. Current Therapeutic Antibodies 

Antibodies are molecules that are highly specific to their targets (see Table 2). This specificity and high-affinity binding are essential for the neutralization of highly toxic toxins. The efficiency of antibodies inactivating RT has been recognized for more than a century [90]. Nevertheless, the neutralizing mechanisms have only been revealed in recent years via the characterization of neutralizing and non-neutralizing mAbs directed against RT’s enzymatic and binding subunits. Using a collection of neutralizing and non-neutralizing mAbs against defined linear epitopes on RTA and RTB, the epitope map of ricin holotoxin was constructed, showing distinct “hot” and “cold” regions that correspond to neutralizing and non-neutralizing epitopes. This information supports the engineering of RTA and RTB mutants that are more effective than native antigens in eliciting protective immunity [91]. In addition to precise epitope mapping, it is essential to select antibodies having the broadest spectrum of neutralizing effects to act against different ricin isoforms. It was proved that targeting RTA, RTB, or the RTA–RTB interface is efficient for ricin neutralization [92]. Several efficient anti-ricin recombinant humanized monoclonal antibodies (mAbs) have been evidenced with the potential to be used for prophylactic or therapeutic purposes against ricin poisoning [93]. Polyclonal antibodies are also still of interest and under development. Moreover, anti-ricin antibodies block ricin′s entry into cells and hinder its intracellular routing, showing that RT can be neutralized intracellularly. In addition, it was shown that the JJX12 antibody was able to disrupt the route of toxin uptake and trafficking within cells by directly cross-linking RT [94]. These mechanistic results support the use of post-exposure antibody therapy [95].

Several antibodies that neutralize ricin have been identified. Here, some examples are reviewed to demonstrate the proof of concept of antibody-based therapy.

PB10 is a murine mAb directed against an immunodominant epitope on the enzymatic subunit of RT. The PB10 neutralizes ricin by interfering with the transport within the trans-Golgi complex network, presumably by driving ricin to lysosomes for its degradation. Because murine antibodies are poorly tolerated, PB10 was chimerized (cPB10) by grafting its variable domains on human IgG1 and K constant domains [96]. Despite its affinity of only 40 nM, the IC_50_ of cPB10 is 0.03 µg·mL^−1^. A single dose of 5 µg of cPB10 fully protected mice from death when it was administered i.p. 24h before an i.p. challenge with 10 LD_50_ of ricin. More interestingly, the cPB10 antibody fully protected BALB/c mice when a single dose of 10 mg·kg^−1^ of cPB10 was administered 24h before, simultaneously, or 4h after an aerosol challenge with ~5 LD_50_ of ricin. Proof of efficacy against an aerosol challenge is interesting because this route of intoxication may be used in a bioterrorist context. The largest therapeutic window is also required because it is probable that a ricin intoxication will not be identified directly by the medical staff. cPB10 was then germline-humanized (hPB10) to increase its tolerance again. This humanization was successful because the identity of the variable domain increased from 70% to 90%. Antibody functions were preserved after humanization. After humanization, the PB10 variable domain’s overall identity increased from 70% to >90% without affecting antibody neutralizing potential. The i.p. administration of 5 or 40 µg of hPB10 24 h before a ricin challenge fully protected mice from death after an i.p. or intranasal challenge with 10 LD_50_ of ricin. However, it was recently demonstrated that the therapeutic capacity of humanized huPB10 IgG1 is significantly improved when co-administered with a second humanized antibody, huSylH3, targeting RTB and having an affinity of 57.4 pM. It was demonstrated that a cocktail composed of huPB10/huSylH3 is superior to PB10 alone when used as a pre-exposure prophylactic in a mouse model of intranasal RT challenge. Mice that were challenged with ricin plus huSylH3 IgG1 (2 mg·kg^−1^) alone died, while mice that received huPB10 alone survived the challenge, although they lost a significant amount of weight on days 3–7. Both antibodies (2 mg·kg^−1^) were administered intranasally to mice before (-48, -24, -4, and -0 h) a challenge with 10 LD_50_ of ricin. All mice receiving the antibody cocktail survived the challenge. Only the group of animals that received the cocktail 48 h before the challenge experienced significant weight loss, but all mice, except one, recovered by day 14. These results demonstrate the capacity of the humanized huSylH3/huPB10 MAb cocktail to neutralize RT in a mouse model of pulmonary toxin exposure. The PB10/SylH3 cocktail was also superior to PB10 alone when used as a pre-exposure prophylactic in a mouse model of the intranasal ricin challenge [97]. These data thereby open the door to future testing in non-human primates [98]. Indeed, it was recently reported that administration of PB10 and SylH3 as an RT-mAb immune complex to mice via intranasal or i.p. routes triggered a rapid onset of ricin-specific serum IgG persisting for months. RIC administration also induced high titers of toxin-neutralizing Abs. In addition, it was confirmed that intranasal RIC administration was more effective than i.p. delivery in inducing immunity against intranasal RT exposure [99].

43RCA-G1 is a recombinant antibody that binds the ricin A chain with an affinity of 40 pM [100]. This antibody was isolated using phage display technology starting from an NHP (Macaca fascicularis) immunized with the non-toxic A chain of ricin. The 43RGA-G1 was then germline-humanized to optimize its tolerance. The 43RCA-G1 neutralized 89% of ricin activity at 40 µg·mL^−1^ and 50% of ricin activity at 1.5 µg·mL^−1^ in an in vitro cell-free neutralizing assay (corresponding to a molar ratio of [43RCA-G1]/[ricin] of 12). In particular, 43RCA-G1 protected cynomolgus monkeys from ricin intoxication after intranasal administration of 24 μg·kg^−1^ when the antibody was nebulized [101]. This antibody nebulization device could be useful for an easy and rapid antibody treatment in the context of a massive biological attack. 

RB34 and RB37 are two picomolar mouse mAbs directed against RCB [102]. The affinity of RB34 and RB37 was determined against ricin D and E. Targeting RTA and RTB with a cocktail of antibodies (polyclonal antibodies) may lead to better neutralization. Therefore, 43RCA-G1, RB34, and RB37 were tested as a cocktail of two or three antibodies against ricin D and/or E from different cultivars. These antibodies target different epitopes and do not compete for the binding to ricin. With single antibodies, partial or low protection from ricin may be observed in some cultivars. A recent study evidenced the effectiveness of the synergistic effect when combining RB34 and 43RCA-G1 antibodies. This synergistic effect was confirmed in vivo in a mouse model of intranasal intoxication with ricin D and E [103]. The combination of RB34 and 43RCA-G1 had the best neutralizing capacity. The IC_50_ of the antibody combination was 2–12 times lower than that of each individual Ab *(p* < 0.01), except for two ricin cultivars. No significant advantage was conferred by the addition of RB37. These data underline that oligoclonal antibodies confer a significant advantage for the treatment of ricin intoxication. Indeed, as ricin intoxication is rare and mortality may occur in the first four days, there is no time for the identification of the type of ricin.

Polyclonal antibodies represent an alternative to monoclonal and recombinant antibodies. For example, Falach et al. [104,105] demonstrated the therapeutic efficacy of equine-derived anti-ricin F(ab’)_2_ antibodies against lethal pulmonary and systemic ricin exposures in a swine model for the first time. Swine were challenged with a dose of crude ricin of 3 µg·kg^−1^ (intratracheal route) or 7.5 mg·kg^−1^ (intramuscular route). Administration of the antitoxin at 18 h post-exposure protected more than 80% of the swine, either intoxicated by intratracheal or intramuscular route. Moreover, treatment at 24 h post-exposure protected 58% of the intramuscularly exposed swine, as opposed to 26% of the intratracheally exposed animals. 

Bispecific antibodies are molecules of particular interest for targeting two different epitopes on a ricin molecule. The JJX12 is a bispecific antibody consisting of RTA-D10, a camelid single-variable domain (VHH). This antibody is directed against an epitope on RTA and linked to RTB-B7, a VHH antibody directed against RTB, via a 15-mer peptide [93]. It was proved that the in vivo mouse protection induced by this bispecific antibody is better than that induced by an equimolar mixture of both antibodies. JJX12 fully protected mice from a ricin challenge with a mixture composed of 10 LD_50_ of ricin and JJX12 incubated 1 h before i.p. injection. Full protection was observed when an antibody–ricin ratio of 4:1 was used. Interestingly, the homodimer of each antibody failed to protect mice from death because the linker was too short target the epitope of two ricin molecules. 

Neutralization of ricin with single-domain antibodies is another approach of interest; however, for the neutralization of toxins, full-length antibodies are recommended to facilitate the fast elimination of the toxin via the recruitment of effector cells. As an example, V5E1 is a VHH that was obtained by screening a camel-derived phage display library. V5E1 binds RCA with an affinity of 20 pM, and in vitro, its IC_50_ is less than 1 nM [106].

## 6. Chemical Inhibitors

Antibodies are very efficient for the neutralization of toxins and generally well-tolerated, particularly when only one or few administrations are required. Nevertheless, antibody production is generally expensive. Chemical inhibitors and small proteins may represent an interesting alternative to antibodies, even though they are potentially more toxic than antibodies. 

Aitbakieva et al. [107] demonstrated that VPg1–110, the N-terminal truncated variant of viral genome-linked protein (VPg) from turnip mosaic virus (TuMV), bound to RTA and abolished ricin’s catalytic depurination of 28S rRNA in vitro and in a cell-free rabbit reticulocyte translational system. In the cell-free rabbit reticulocyte translational system, the treatment of RTA with the synthetic VPg1–110 peptide resulted in complete inhibition of RTA activity on ribosomes.

The search for molecules that block ricin intracellular trafficking has evidenced compounds that mostly target the Golgi apparatus. However, these molecules’ side effects preclude their therapeutic development. One example of such an agent is a sesquiterpenoid quinone metabolite isolated from marine sponges, namely, ilimaquinone. The ilimaquinone blocks the cytotoxicity of ricin in Vero cells in a dose-dependent manner by disruption of the Golgi apparatus and its fragmentation into small vesicular structures [108,109]. Similarly, brefeldin A (BFA), an isoprenoid fungal metabolite, inhibits ricin toxicity in vitro by dismantling the structure and function of the Golgi apparatus [110,111]. However, BFA irreversibly impairs intracellular protein transport and secretion and sensitizes some cell lines to ricin (MDCK and PtK2 kidney epithelial cell lines) [112]. BFA was fully characterized approximately 40 years ago following its identification as a natural compound. As mentioned by Barbier and co-authors, the elucidation of its mechanism of action has given birth to the general concept of interfacial inhibition. This inhibitor traps macromolecules as a dead-end complex, which then becomes unable to complete their toxic role via interaction with the host interfaces. 

Two other inhibitors, namely, Exo 2 and its derivative LG186, have been identified as inhibitors of secretory pathways. Exo 2 rapidly inhibits anterograde traffic to the Golgi apparatus, disrupting ricin trafficking [113,114].

Several toxins, such as ricin, cholera toxin, and Shiga toxin, have the capacity to undergo retrograde transport from the plasma membrane to the endoplasmic reticulum. Retro-2 is a small chemical compound that neutralizes toxin activity by inducing toxin accumulation in early endosomes and relocalization of the Golgi SNARE protein syntaxin-5 to the endoplasmic reticulum [115]. More precisely, Retro-2 targets the endoplasmic reticulum exit site component Sec16A, affecting the anterograde transport of syntaxin-5 from the endoplasmic reticulum to the Golgi. The principal advantage of Retro-2 is that it neutralizes the toxin activity by targeting its intracellular pathway instead of targeting the toxin itself. Due to this mechanism, Retro-2 has a broad-spectrum activity that has been evidenced both in vitro and in vivo against ricin, Shiga toxin-producing entero-hemorrhagic *E. coli* and *Leishmania sp*., and in vitro against Ebola virus, Marburg virus, poxviruses, and Chlamydiales [116,117]. Retro-2 was characterized in a model of ricin intoxication via nasal instillation of a dose of ricin, leading to 90% deaths by day 21 (LD_90_) [116]. With this dose, the first clinical signs of toxicity were observed within 24 h. Statistically significant prophylactic protection was observed in the first experiments with a single i.p. dose of 2 mg·kg^−1^ of Retro-2 one hour prior to toxin challenge: 49% of the mice survived vs. 11.5% in the control group (*p* = 0.001). After administration of 20 mg·kg^−1^ and 200 mg·kg^−1^, the survival was 60% and 100% at 20 days, respectively. Retro-2 was solubilized in DMSO, and no toxicity was observed in animals after i.p. administration up to 400 mg·kg^−1^. Retro-2 and its derivative Retro-2.1 represent drugs of choice for further development.

Some natural molecules have also been described to inhibit ricin toxin activity. The catechin epigallocatechin gallate (eGCG), found in green tea, neutralizes ricin activity [118]. In vitro, in a cell-based assay (Vero cells and THP-1), the addition of 100 µM of eGCG significantly reduced the activity of ricin. It was hypothesized that eGCfvG could, directly or indirectly, modify the conformation of the ricin B chain and reduce its affinity for the ricin receptor at the cell surface.

## 7. Development of Anti-Ricin Vaccines

Generally, the best approaches to prevent a pathology are prophylaxis and, in particular, vaccination. Because of the cost and the balance between the benefit and the risk of a vaccine, vaccination is generally not recommended for rare diseases or toxins. In the context of toxins, post-exposure vaccination is generally not efficient. In studies with rhesus macaques, at 4 h post-intoxication, administration of a neutralizing mAb protected 5/5 monkeys, but only 1/5 at 12 h post-intoxication [119]. Considering the risk of bioterrorism and due to the potential short window for treatment, a vaccine may be suitable in some contexts, and anti-ricin vaccines are currently being studied. In a normal context, anti-ricin vaccination could be limited to people at high risk of exposure (scientists, first responders, or soldiers) to prevent laboratory accidents.

Previous studies have shown the A chain to be more immunogenic than the B chain [120]. Thus, two recombinant vaccines based on immunization with the RTA subunit are currently in phase 1 and 1B development, namely, RVEcTM (US Army Medical Research Institute of Infectious Diseases: USAMRIID, Frederick, MD, USA) and RiVax^®^ (the University of Texas Southwestern Medical Center, Dallas, TX, USA) [121]. 

Rivax is an alum-adjuvanted subunit vaccine candidate that may prevent death and injury from any route of exposure to RT. A major advantage of this vaccine is that RiVax^®^ is a thermostabilized vaccine candidate that can be stored at room temperature for extended periods and is stable up to 40 °C. Such stability is essential for stockpiling and in a military context because soldiers may be engaged everywhere in the world and because the cold chain may not be respected. RiVax^®^ is composed of a modified form of the A chain of RT that removes the biological activity of the protein while still retaining its shape to trigger an effective antibody response. One mutation disrupts the ribotoxic site (Y80A), and the other one disrupts the vascular-leak-syndrome-inducing site (V76M) [122,123]. RiVax^®^ is administered as an intramuscular injection on two or three occasions, resulting in the adaptive immune system mounting an antibody response. In a clinical study, all volunteers with anti-RTA antibodies also had ricin-neutralizing antibodies in their sera 2 weeks after the third vaccination. It was observed that the level of neutralizing antibodies was not related to the dose of the vaccine given, but almost all volunteers receiving the lowest vaccine dose did not develop an antibody response. An individual whose antibody had the most robust neutralizing activity received their third vaccination 112 days after the second vaccination, whereas all the other volunteers received theirs 28–42 days after the second vaccination. A passive protection assay was realized in a mouse model with the sera of the volunteers that developed neutralizing antibodies. The highest doses tested (62.5 μg and 25 μg) protected the mice from 5 LD_50_ of ricin (or 2 μg), whereas lower doses (12.5 μg and 5.0 μg) did not. The RiVax^®^ antigen has been shown to be safe in two phase 1 studies in humans. None of the 15 volunteers experienced grade 3 or 4 symptomatic toxicity in this study. Two experienced grade 2 toxicities. The toxicities were those often associated with i.m. injections of approved vaccines. The development of RiVax^®^ is pursuing its approval under the Animal Rule. Future steps will include pivotal animal efficacy studies (to demonstrate potency in animals) and phase 2 clinical studies in humans (to confirm its safety and correlate immune markers of protection with outcomes from animal studies).

A recombinant ricin vaccine from E. coli (RVEc™) was developed at the USAMRIID (Frederick, MD, USA) and assessed in an FDA-sponsored phase 1a clinical trial. RVEcTM is a truncated derivative of RTA that lacks the hydrophobic carboxy-terminal region (residues 199–267) as well as a small hydrophobic loop in the N-terminus (residues 34–43), resulting in a molecule with increased solubility and thermal stability [124,125,126]. RVEc^TM^ does not contain mutations that directly inactivate the active site of RTA, but the removal of both segments results in an inactive molecule devoid of enzymatic activity and a reduction in or elimination of its ability to cause a vascular leak, as demonstrated in experimental models. RVEc^TM^ is adjuvanted with Alhydrogel^®^. Volunteers received 3 injections at 4-week intervals with 20 or 50 μg of vaccine (n = 10 for each group), and a group of volunteers received a single dose of 100 μg. RVEc™ was safe and well-tolerated at all doses. The most common adverse events were pain at the injection site and headache. Of the 10 subjects who received a single 100 μg dose, 2 developed elevated creatine phosphokinase levels, which resolved without sequelae. Anti-ricin IgG titers of 1:500 to 1:121,500 were observed using ELISA assays in subjects immunized with 20 µg or 50 μg. A total of 50% of them produced neutralizing anti-ricin antibodies measurable via TNA. Four subjects in the 50 μg group received a single booster dose of RVEc™ 20–21 months after the initial dose. The single booster was safe and well-tolerated, resulting in no serious adverse events and significantly enhanced immunogenicity of the vaccine in human subjects. Each booster recipient developed a robust anamnestic response with ELISA anti-ricin IgG titers of 1:13,500 to 1:121,500 and neutralizing antibody titers of 1:400 to 1:3200.

## 8. Concluding Remarks and Perspectives

The state of the art of medical countermeasures against ricin shows the multiple promising strategies for the treatment and prevention of ricin poisoning. All of them have advantages and disadvantages in terms of efficiency, cost, tolerance, and therapeutic window.

The clinical development of medical countermeasures against ricin is quite complex because ricin intoxications are rare and not of particular interest to pharmaceutical companies, except when states agree to stockpile such medical countermeasures in the context of a national biological weapon prevention program. Additionally, clinical phase 2 or 3 trials would be complex because of the very low number of human cases each year. Therefore, clinical development would be based on the FDA Animal Rule. Considering this fact, it is essential, if possible, to produce the molecules of interest under GMP conditions as early as possible and to realize pre-clinical and clinical phase 1 trials when possible.

Comparison of the Advantages and Disadvantages between Medical Countermeasures

The pre-exposure vaccination approach will elicit the production of neutralizing antibodies that inactivate ricin in vivo, but a mass vaccination program to protect whole populations against ricin remains unrealistic due to the low probability of being exposed to ricin compared to the potential adverse effects of the vaccine, as well as the cost of vaccination. Post-exposure vaccination has not been deeply studied in the context of ricin, but generally, such an approach demonstrates no or poor protection against non-infectious pathogens. Neutralizing antibodies are the most specific drugs, and several of them proved their efficacy in animal models. Inhibition of the catalytic activity of the enzymatic moiety of ricin by small-molecule compounds has been confirmed in in vitro tests, but such inhibitors were unable to protect cells or animals against a ricin challenge, and the molecules altering the intracellular trafficking of ricin showed dramatic effects on the Golgi apparatus, precluding their therapeutic development.

The following is a summary of the available countermeasures: Ricin post-exposure treatment should include an antitoxin as the first line of treatment. Small-molecule inhibitors may be an alternative if no antibodies are available. In addition to toxin-specific countermeasures, anti-inflammatory or immunomodulatory agents as well as regenerative therapies will optimize the recovery of the cell machinery. The more recent strategies to tackle the risk of exposure to ricin include chimeric neutralizing antibodies, humanized antibodies, and promising bispecific mAbs [127].

Since promising antibody and vaccine options are currently available, it is essential to guarantee stockpiles for future use. The next challenge is thus to adapt government policies to allow access to such countermeasures in case of a mass biothreat attack.

## Figures and Tables

**Table 1:** Presentation of a selection of techniques used for the detection of ricin. The limit of detection is lower than the limit of quantification and depends on the matrix.

Detection Method	Limit of Detection	Specificity	Reference
Mass spectrometry	Up to 0.2 ng·mL^−1^	Low to high (depending on the selected peptides)	Kalb and Barr, 2009
RTA activity assays	0.1–25 ng·mL^−1^		Brigotti et al., 1998; Heisler et al., 2002 Becher et al., 2007; Bevilacqua et al., 2010 Haes et al., 2006; Kirby et al., 2004; Lamont et al., 2011
Multiple reaction monitoring (MRM) mass spectrometry (MS) and electrospray ionization tandem mass spectrometry (LC-ESI-MS/MS)	2.5 ng·mL^−1^	High	Ma et al., 2014 Schieltz et al., 2015
MALDI TOF MS-based activity assay	0.2 ng·mL^−1^	High	Wang et al., 2016
Ricin capture coupled with benchtop MALDI-TOF MS mass spectrometer	8 ng·mL^−1^	High	Hoyt et al., 2021
ELISA assays	10–14 ng·mL^−1^	High	Dayan-Kenigsberg et al., 2008; Fulton and Thompson, 2007
Fluorescence-based fiber optics, gold particles, or electrochemiluminescence	100 ng–100 pg·mL^−1^		Narang et al., 1997; Shyu et al., 2002b; Garber and O′Brien, 2008; Simon et al., 2015; Yu et al., 2000; Shankar et al., 2005
Microspheres	5 ng·mL^−1^		
Capture with DNA/RNA aptamers, single-domain antibodies, or sugar-conjugated materials.	14–30 ng·mL^−1^		Haes et al., 2006; Lamont et al., 2011
Surface plasmon resonance (SPR), polymerase chain reaction (PCR), or mass spectrometry (MS) coupled to enrichment steps	1 fg·mL^−1^ 0.093 ng·mL^−1^		Anderson et al., 2013; Blome et al., 2010; Nagatsuka et al., 2013; Uzawa et al., 2008; He et al., 2010; Lubelli et al., 2006; Duriez et al., 2008; Pittman et al., 2013; Roen et al., 2013; Kanamori-Kataoka et al., 2011; Chen et al., 2014
Cell-based assays	0.3 ng·mL^−1^	Low to medium	Pauly et al., 2012; Makdasi et al., 2019
RIPpore assay with direct RNA sequencing	0.9 ng	Medium	Ryan et al., 2022

**Table 2:** Example of antibodies that neutralize ricin toxin. The affinity for ricin D and E may be different, but the affinity for both isotypes is generally not determined.

Antibody Types	Antibody Name	Affinity	Reference
Murine monoclonal	PB10, RB34, RB37	PB10: 40 nM, RB34 and RB37: 150 pM and 224 pM, respectively, against whole ricin. RB34: 10 pM (ricin D) and 5.42 nM (ricin E), RB37: 137 pM (ricin D).	Sully et al., 2014, Prigent et al., 2011, Orsini Delgado et al., 2021
Humanized monoclonal	cPB10, huPB10, huSylH3 (alone or in combination), 43RCA-G1	43RCA-G1: 48 pM (ricin D and E). huSylH3 (0703): 57.4 pM, cPB10 and huPB10: 40 nM.	Hu et al., 2012, Rong et al., 2020a and b, Tolman et al., 2022; Pelat et al., 2009; Respaud et al., 2016; O’Hara et Mantis 2010
Polyclonal and multispecific antibodies	JJX12 (bispecific), horse-derived polyclonal F(ab’)2 (RR-001 and RR-002)	Bispecific JJX12: 630 pM (RTA-D10 part) and 1330 pM (RTB-B7 part). Polyclonal: not applicable.	Herrera et al., 2016; Falach et al., 2020; Vance and Shoemaker 2011
Camel-derived nanobodies (phage display V_H_Hs)	V5E1	20 pM	Rudolph et al., 2021 and 2017

Note: For some antibodies, the authors did not specify if the affinity is for ricin D or E. The affin-ity for ricin D and E from different cultivars may be different. The affinity can also be influenced by the system used to determine it (Biacore, Octet, etc.) and could not be rigorously compared. The affinity may be different after the expression of an antibody fragment in the format of a full-length antibody.

## Data Availability

Not applicable.
